# Refined cuff technique minimizes surgical complexity in rat lung transplantation and improves outcomes

**DOI:** 10.1038/s41598-025-19711-2

**Published:** 2025-10-14

**Authors:** Mehmet Furkan Sahin, Gunes Esendaglı, Fatma Yıldırım, Erdal Yekeler, Banu Kılıcaslan

**Affiliations:** 1 Department of General Thoracic Surgery and Lung Transplantation, Ankara Bilkent City Hospital, University of Health Sciences, Ankara, Turkey; 2 Department of Basic Oncology, Cancer Institute, Hacettepe University, Ankara, Turkey; 3 Department of Pathology, Faculty of Medicine, Lokman Hekim University, Ankara, Turkey; 4 Department of Anesthesiology and Reanimation, Faculty of Medicine, Hacettepe University, Ankara, Turkey; 5 Department of Thoracic Surgery and Lung Transplantation, University of Health Sciences Ankara Bilkent City Hospital, Universiteler, 1604. Cd. No: 9, Cankaya, Ankara, 06800 Turkey

**Keywords:** Lung transplantation, Cuff technique, Model modification, Rat, Anastomosis, Diseases, Engineering, Medical research

## Abstract

Orthotopic lung transplantation in rats serves as a pivotal platform for exploring long-term allograft outcomes, yet technical complexity has limited its widespread application. Here, we present a refined surgical model that combines structured intraoperative modifications with tailored mechanical ventilation strategies to ensure procedural consistency and biologic fidelity. Key interventions—such as a custom leak-proof endotracheal tube, a temporally controlled reperfusion maneuver, and an optimized anastomotic sequence—collectively addressed critical challenges including airway obstruction, graft impaired perfusion, and venous fragility. The protocol demonstrated a high procedural success rate and reproducible long-term survival, with histopathological analysis confirming progressive chronic remodeling and diminished acute rejection. Importantly, the technique demonstrated features that facilitate rapid acquisition, supported by a short learning curve, and was implemented using widely accessible clinical-grade materials. Together, these attributes establish a robust and scalable platform for investigating chronic lung allograft dysfunction and other long-term immunologic phenomena.

**Supplementary Information:**

The online version contains supplementary material available at 10.1038/s41598-025-19711-2.

## Introduction

Lung transplantation is widely regarded as the most effective therapeutic option for end-stage parenchymal lung diseases, offering both a survival benefit and improved quality of life. Despite its potential benefits, the procedure remains highly complex, riddled with uncertainties, and stands as one of the most critical areas requiring further refinement and improved clinical outcomes. The principal metrics of success in lung transplantation are long-term survival and quality of life. The major obstacle to success stems from the complexity of the adaptive immune system, which rapidly activates to reject the allograft once perfusion is re-established, even though transplantation is performed between genetically compatible individuals (i.e., allotransplantation). Consequently, long-term graft success depends upon maintaining a delicate immunological equilibrium under strict immunosuppressive control. Another critical obstacle to long-term success is the inevitability of chronic rejection, which remains difficult to prevent even under continuous immunosuppressive therapy. For these reasons, lung transplantation is considered the most complex surgical procedure among all solid organ transplants, characterized by both the lowest median post-transplant survival (7.8 years) and numerous unresolved mechanistic aspects^1,2^.

Substantial global efforts are underway to enhance transplant outcomes, with particular emphasis on elucidating the immune mechanisms underlying rejection and tolerance. In clinical settings where causal relationships are often difficult to establish, there is a clear need for in vivo models that simulate human physiology, particularly for ethically untenable interventions such as withholding immunosuppression to examine allograft-specific responses^3^. While samples such as bronchoalveolar lavage fluid or serum can be obtained from human recipients, tissue sampling directly from the allograft—the presumed epicenter of immune activity—is typically precluded unless mandated by clinical urgency. Therefore, experimental animal models that allow assessment of the transplanted organ and its immune interactions are indispensable to developmental and translational investigations aimed at improving graft outcomes.

Most human immune cell analogs have been identified in mice and rats^4^. Nevertheless, the implementation of surgical protocols in small animal models presents substantial technical challenges, requiring considerable skill and expertise. Among available small-animal models, rats are frequently favored over mice due to their slightly larger size, which facilitates surgical manipulation and reduces intraoperative complexity^5^.

The “cuff technique,” originally described by Mizuta et al. in 1989, has since served as the cornerstone of nearly all experimental lung transplantation studies in rats^6^. Despite the decades since its introduction, only a limited number of researchers have succeeded in performing the procedure efficiently within optimal timeframes, and some have contributed valuable refinements to the existing technique^5,7–18^. Despite these commendable efforts, a universally accepted and optimized surgical protocol for this challenging procedure has yet to be established^4^. Furthermore, many previous studies focusing on technical development have recorded primarily intraoperative parameters, with animals typically sacrificed in the early postoperative phase. As a result, long-term survival data, essential for evaluating rejection mechanisms and overall transplantation success, remain limited^7,8^.

In our study, which focuses on advancing surgical methodology, we systematically evaluated numerous previously described modifications of the cuff technique. By identifying both the strengths and limitations of existing protocols, we developed novel technical modifications with the potential to overcome intraoperative challenges, achieve high procedural success rates, shorten the learning curve, and facilitate long-term graft survival. With our meticulously detailed surgical approach, we demonstrate that this demanding procedure can be performed successfully using readily accessible instruments, without requiring highly advanced laboratory infrastructure or intensive postoperative care. Ultimately, we aim to encourage researchers to adopt this accessible and replicable model as a robust platform for translational studies in lung transplantation.

## Methods

### Study design

This study was designed as an exploratory investigation aimed at the development and refinement of surgical techniques in rodent lung transplantation, and was approved by the local institutional animal ethics committee prior to initiation (see Ethical Statement). All procedures involving animals were conducted in accordance with relevant institutional and national guidelines and regulations. This study has been reported in compliance with the ARRIVE guidelines (https://arriveguidelines.org) to ensure transparency and reproducibility in animal research. The primary endpoint was defined as the ability to successfully complete the surgical procedure and maintain recipient survival for at least seven days post-transplantation, allowing assessment of acute outcomes such as primary graft dysfunction and early immunological events. As a secondary objective, long-term survival beyond one month was pursued to facilitate evaluation of chronic rejection mechanisms.

To ensure methodological consistency, all surgeries were conducted by a single experienced surgeon under conditions that, while not strictly sterile, adhered as closely as practicable to accepted standards of cleanliness. The operator is a board-certified thoracic surgeon with over a decade of clinical experience, actively involved in human lung transplantation. Prior to this study, the operator had no formal microsurgical training or experience with small-animal transplant models, aside from practicing lung separation and cuff preparation on six explanted rat lungs to evaluate procedural feasibility. Both donor and recipient animals were outbred male Sprague Dawley rats. Donors were 8–10 weeks old and weighed 230–280 g, whereas recipients were 10–12 weeks old with a comparable weight range of 230–280 g. Only animals meeting these predetermined inclusion criteria were enrolled; those failing to meet age, sex, or weight requirements were excluded from the study. Study animals were housed under standardized environmental conditions (22 ± 2 °C, optimal humidity, 12-hour light/dark cycle) at the Kobay Experimental Animals Laboratory (Ankara, Türkiye), maintained on a standard diet with free access to water, and fasted for eight hours prior to surgery.

A total of 40 orthotopic left lung transplantations were conducted. The initial 13 procedures employed various previously described modifications of the cuff technique, primarily based on approaches illustrated in published literature and widely circulated surgical videos^7,8,11^, which proved particularly valuable during the early learning phase. These cases constituted the “Initial Experience” (IE) group, representing the early phase of technical development. During this learning process, each factor contributing to procedural prolongation or suboptimal outcomes was carefully analyzed prior to subsequent cases. Through iterative refinement, the 14th case marked the first successful implementation of all developed modifications. Following this milestone, all remaining transplantations were performed using the fully refined technique, and the corresponding data were analyzed under the “Refined Experience” (RE) group (*n* = 27).

The adopted grouping methodology was intended to enable a rigorous assessment of how the procedural learning curve and cumulative technical modifications impacted operative performance and outcome metrics.

Intraoperative time intervals were defined as follows:

- Donor procedure time: From midline incision to procurement of the heart-lung block.
- Organ preparation time: From placement of the graft in a Petri dish to completion of cuff placements.
- Recipient procedure time: From skin incision (in right lateral decubitus) to initiation of graft reperfusion.
- Total procedure time: From donor skin incision to closure of the recipient’s thoracotomy.

Importantly, recipient and total procedure times were calculated only in cases where graft implantation was successfully completed, and thoracic closure was achieved.

Postoperative monitoring included oxygen saturation measurements performed at full recovery from anesthesia (approximately one-hour post-extubation). Recipients that recovered from anesthesia were monitored daily for survival duration without adjunct immunosuppressive therapy, receiving standard nutrition and hydration *ad libitum*. Post-transplant care included intramuscular analgesia (meloxicam) and thermal support throughout the early postoperative period. Rats surviving to postoperative day seven or longer were euthanized under general anesthesia, and intrathoracic findings were systematically documented. Euthanasia was performed under deep anesthesia by intraperitoneal administration of pentobarbital sodium at a dose of ≥ 200 mg/kg, in accordance with institutional protocols.

For long-term evaluation, three recipients were permitted to survive beyond the acute phase (> one month) and were sacrificed at two, four, and six months post-transplantation to assess time-dependent histopathological changes associated with chronic rejection. Additionally, one sham-operated animal (thoracotomy only) was sacrificed at six months as a control. All tissue samples were assessed by an independent pathologist under single-blinded conditions.

Prior to euthanasia, 0.5 mL of blood was collected from the tail vein into EDTA tubes for complete blood count analysis, enabling comparison between long-term and one-week survivors. The detailed allocation of animals, the analysis-specific n values, and the group-specific exclusions are summarized in Table 1 for clarity.

**Table 1:** Summary of analytical endpoints, sample sizes, and exclusions per group.

Analysis type	IE Group (*n*)	RE Group (*n*)
Surgical success rate	7/13	26/27
Postoperative survival to day 7	0/13	26/27
Procedure time analyses (recipient/total)*	13 (7)	27 (26)
Oxygen saturation at 1 h post-operative	7	26
Gross pathology at endpoint	7 (postmortem)	26 (≥ day 7, euthanized)
Long-term observation (≥ one month)	0	3
Histopatological comparison at endpoint	0	3
Hematological comparison at endpoint**	0	23 (one-week) / 3 (long-term)

* Recipient/Total Procedure time analyses were limited to cases in which implantation and thoracic closure was successfully completed.

** Hematological profiles obtained prior to sacrifice were analyzed to identify differences between one-week and long-term survivors.

### Reagents and devices

**Fig. 1:**
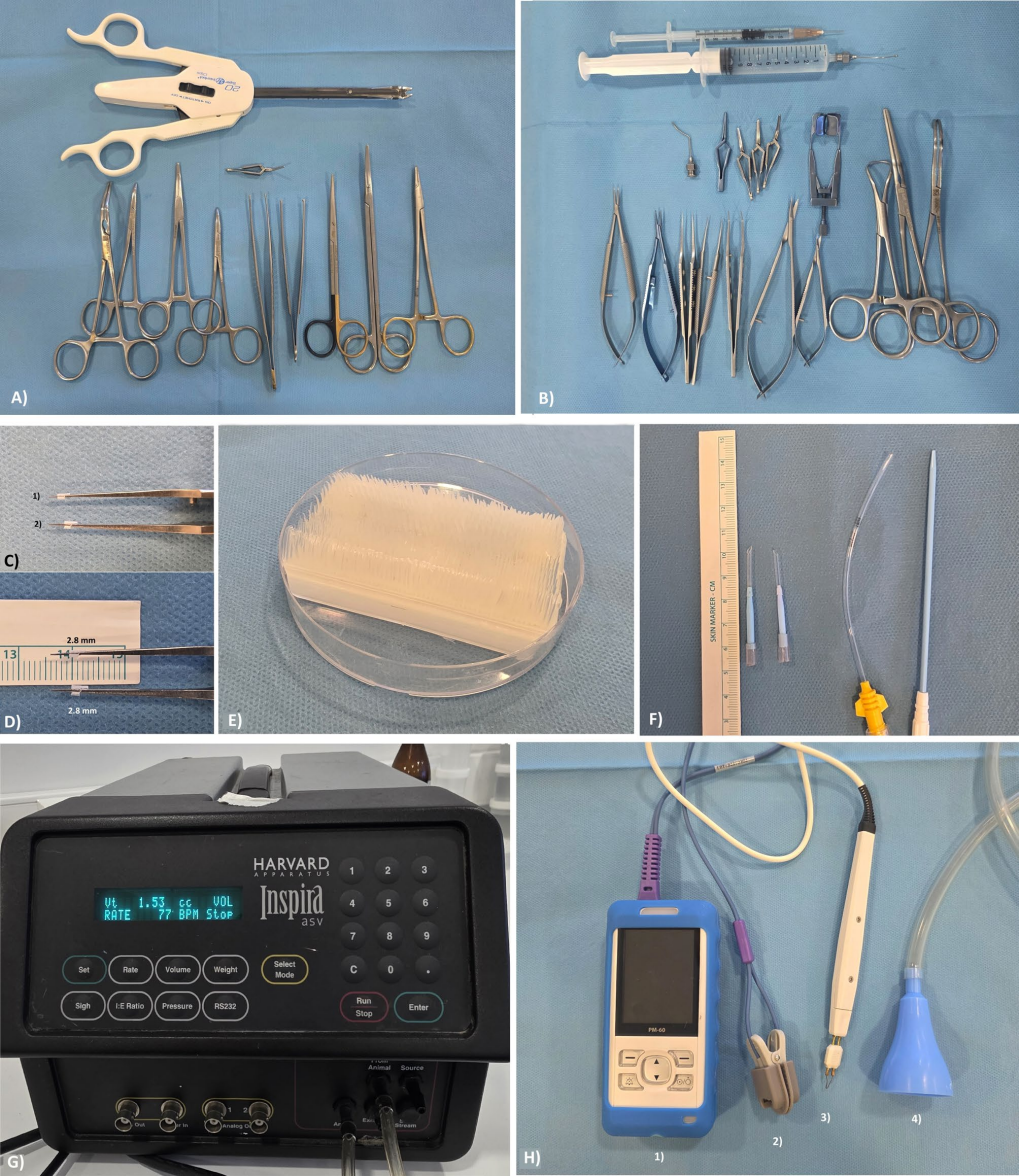
Representative materials and devices utilized during rat orthotopic lung transplantation. (**A**) Donor procedure instruments, (**B**) Recipient-side microsurgical instruments adapted from standard cataract surgery kits; (**C**) Cuff components: 18G green catheter (1) for arterial anastomosis; 16G gray catheters (2) for venous and bronchial anastomoses; (**D**) Standard cuff design (1.4 mm body and 1.4 mm tail length); (**E**) Soft-bristled brush positioned in a Petri dish, providing protective cushioning during graft preparation; (**F**) Custom-designed endotracheal tube fashioned from a central venous catheter for atraumatic intubation; (**G**) Harvard Apparatus^®^ Inspira ASV ventilator used for controlled mechanical ventilation of small animals; (**H**) Monitoring and support devices: (1,2) Pulse oximeter with rodent-adapted probe; (3) Electrocautery unit for intraoperative hemostasis; (4) Improvised respiratory mask repurposed from a clinical drainage funnel for postoperative oxygen delivery.

All microsurgical procedures were performed under 10× magnification utilizing a stereoscopic microscope (RF4^®^-7050, Guangzhou, China). The majority of microsurgical instruments employed were adapted from ophthalmic microsurgical kits routinely used in human cataract operations (see Fig. 1A, B). A small animal mechanical ventilator (Harvard Apparatus^®^ Inspira ASV, USA) was used to provide volume- and pressure-controlled ventilation, with automatic calibration of tidal volume and respiratory rate based on animal weight (see Fig. 1G).

Endotracheal intubation was achieved via a custom-designed tube tailored for rat tracheal dimensions. This device was constructed by modifying the dilator component of a standard central venous catheter, cut to a 2 cm segment and augmenting it with a similarly sized portion of the catheter’s flexible serum channel. The tracheal end was beveled to facilitate atraumatic insertion through a minimal tracheostomy (see Fig. 1F).

Heparin sodium served as the anticoagulant agent throughout the procedure. For graft preservation, 10 mL of low-potassium dextran (LPD; Perfadex^®^, Sweden), supplemented with 20 IU/mL heparin sodium, was administered. To minimize the risk of parenchymal trauma during donor lung separation and cuff preparation phases, a soft-bristled brush was placed within a Petri dish to provide cushioning support for the graft (see Fig. 1E).

Anastomotic cuffs were designed as 1.4 mm body and 1.4 mm tail length (see Fig. 1D). Sixteen-gauge gray intravenous catheters were selected for the bronchus and pulmonary vein, while 18-gauge green catheters were designated for the pulmonary artery (see Fig. 1C). For cuff fixation and assembly, suture materials included 6 − 0 silk and 8 − 0 Prolene^®^, for thoracic closure 4 − 0 Prolene^®^, and for tracheostomy repair 6 − 0 Vicryl^®^ was used. Hemostasis during the procedure was achieved using electrocautery (see Fig. 1H3). Postoperative oxygen saturation was monitored using a rodent-adapted pulse oximeter probe (see Fig. 1H1,2).

During the recovery phase, high-flow oxygen support was provided using improvised respiratory masks fashioned from clinical drainage bottle funnels, repurposed to conform to the rat’s facial anatomy (see Fig. 1H4). Complete blood counts were conducted using the Mindray^®^ BC-30 Vet hematology analyzer (Shenzhen, China).

### Surgical technique

#### Donor procedure

**Fig. 2:**
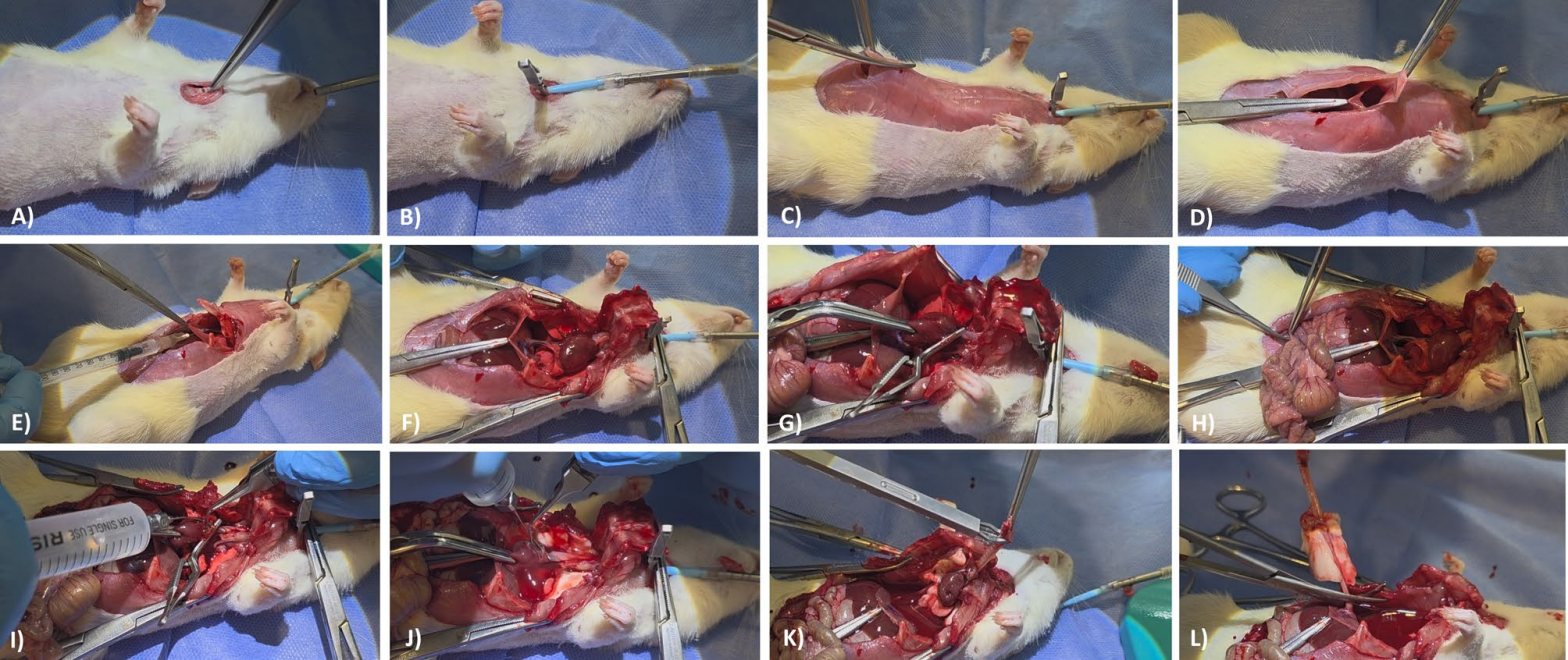
Stepwise overview of donor lung procurement in the rat orthotopic lung transplantation model. (**A**) Tracheostomy via minimal transverse incision on the anterior tracheal surface; (**B**) Endotracheal intubation using a custom-designed catheter-based tube tailored for rats; (**C**) Midline laparotomy to access the abdominal cavity; (**D**) Diaphragmatic incision just above the xiphoid process for bilateral thoracic access; (**E**) Intravenous injection of heparin sodium (1,000 IU/kg) via the inferior vena cava (IVC); (**F**) Thoracic exposure optimized through sternal and costal retraction; (**G**) Clamping of the left atrial auricle with bulldog forceps followed by tip excision; (**H**) Transection of the abdominal and thoracic IVC segments to initiate exsanguination; (**I**) Infusion of cold organ preservation solution [Low-potassium dextran (LPD) with 20 IU/mL heparin] via pulmonary artery cannula; (**J**) Visual confirmation of successful perfusion by uniform lung blanching; (**K**) Occlusion of the tracheal orifice using an automatic clip post-intubation tube removal; (**L**) En bloc excision of the heart-lung block and transfer into chilled LPD solution for preservation. IVC – Inferior vena cava; LPD – Low-potassium dextran.

General anesthesia was induced in donor rats via intraperitoneal injection of ketamine (75–100 mg/kg) and xylazine (5–10 mg/kg). Once respiratory depth diminished, the rat was positioned supine, and a tracheostomy was performed via a minimal transverse incision on the anterior cartilaginous surface of the trachea, carefully preserving the lateral cartilage rings and the posterior membranous wall (see Fig. 2A). Endotracheal intubation was achieved using a custom-designed tube and connected to a small-animal ventilator (see Fig. 2B).

Mechanical ventilator settings included a target tidal volume calculated as 7.2 mL/kg, with a positive end-expiratory pressure (PEEP) of 2 cmH₂O and a respiratory rate of 60–80 breaths per minute^7^. A midline laparotomy was performed by incising the abdominal musculature and peritoneum to enter the peritoneal cavity (see Fig. 2C). The thin diaphragm was grasped just above the xiphoid process with forceps, and the thoracic cavity was accessed bilaterally without injuring the lungs (see Fig. 2D). The inferior vena cava was identified below the heart, and 1,000 IU/kg heparin sodium was administered intravenously via a transdiaphragmatic injection using an insulin syringe (see Fig. 2E). The sternum and costal margins were retracted to optimize exposure (see Fig. 2F).

The thymic tissue overlying the great vessels was excised to provide clear visualization. A bulldog clamp was placed on the auricle of the left atrium, and the auricular tip distal to the clamp was excised (see Fig. 2G). The intestines were retracted caudally to expose and transect the abdominal portion of the inferior vena cava (see Fig. 2H), after which the clamp on the right auricle was released. The thoracic segment of the inferior vena cava was also transected.

The pulmonary artery at the right ventricular outflow tract was exposed, and a small incision was made using micro-scissors. A cannula was advanced into the artery, and 10 mL of organ preservation solution (maintained at + 4 °C and supplemented with 20 IU/mL heparin sodium) was perfused slowly (see Fig. 2I) and under visual control, ensuring uniform blanching of the lungs (see Fig. 2J).

To prevent air leakage, the trachea was grasped en-bloc, the endotracheal tube was removed, and the tracheal orifice was occluded with an automatic clip applier (see Fig. 2K). The heart-lung block was then carefully dissected from surrounding tissues (see Fig. 2L) and transferred to a Petri dish filled with chilled organ preservation solution. All procedural steps related to donor surgery were recorded and are available in Supplementary Video 1.

### Lung separation

**Fig. 3:**
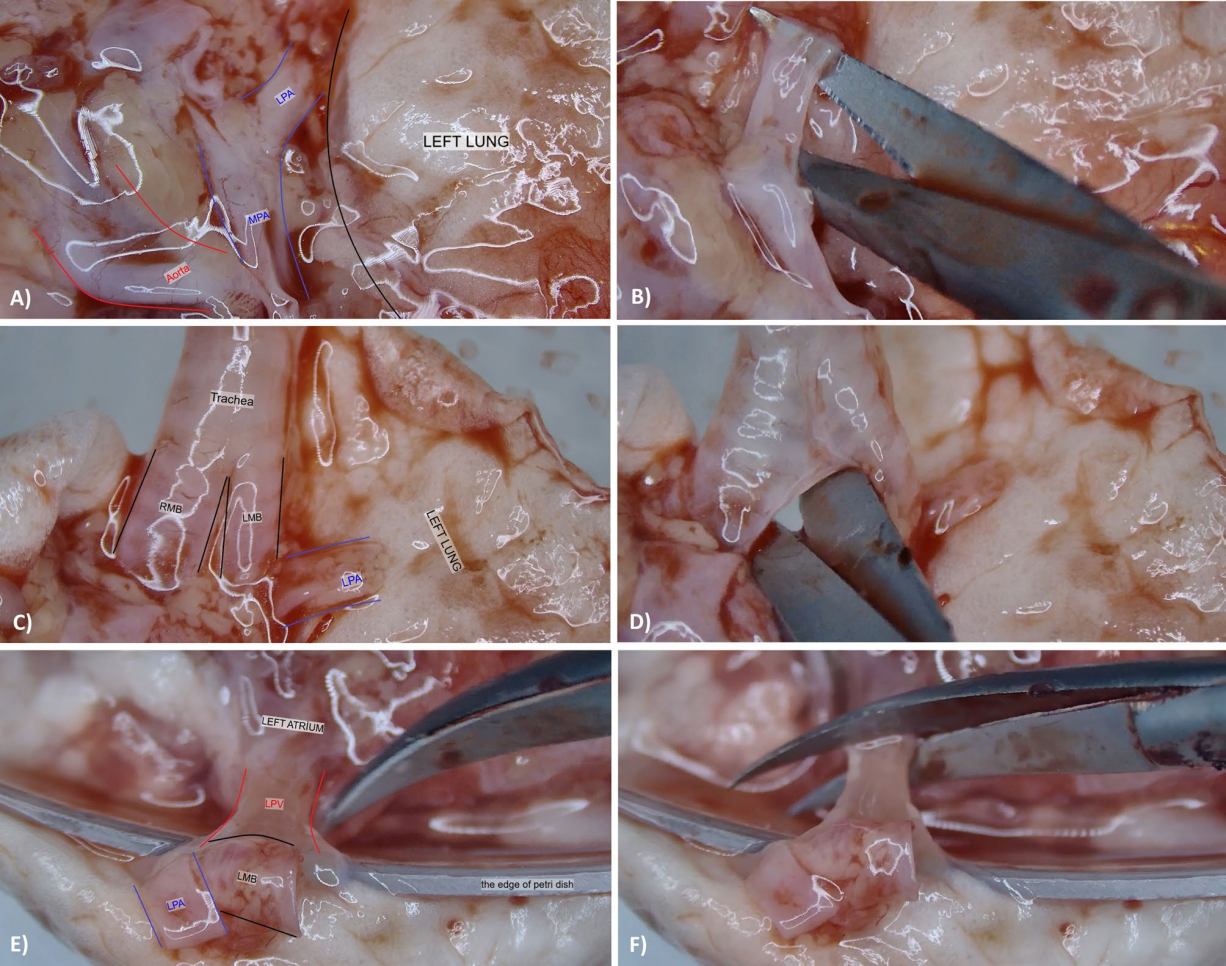
Sequential dissection steps for hilar structure isolation during lung separation. (**A**) Exposure of the left pulmonary artery (LPA) at its origin from the main pulmonary artery (MPA); (**B**) Transection of the LPA just distal to its bifurcation to preserve adequate cuff length; (**C**) Identification and exposure of the left main bronchus (LMB); (**D**) Transection of the LMB below the carina, ensuring sufficient length for anastomosis; (**E**) Posterior approach using the Petri dish edge as a retractor for optimal visualization of the pulmonary venous junction; (**F**) Transection of the left pulmonary vein (LPV) from the left atrium, preserving maximal length for cuff preparation. LPA – Left pulmonary artery; LPV – Left pulmonary vein; LMB – Left main bronchus; RMB – Right main bronchus; MPA – Main pulmonary artery.

The esophagus was first fully separated from the posterior surface of the trachea. All subsequent steps were performed under 10× magnification using a surgical microscope. Residual thymic tissue, adjacent mediastinal structures, and the aortic arch were dissected via an anterior approach. Following this, the origin of the pulmonary artery from the heart was visualized (see Fig. 3A). Dissection proceeded inferior to the bifurcation, favoring the anatomically shorter left pulmonary artery, which was isolated from the main trunk (see Fig. 3B). Next, the trachea and carina were freed from adjacent tissues (see Fig. 3C). Through continued anterior dissection, access was gained beneath the carina, allowing transection of the left main bronchus at a level preserving sufficient length for subsequent anastomosis (see Fig. 3D).

The pulmonary vein was separated from the left atrium as the final step. At this stage, deviating from conventional techniques described in the literature, a posterior approach was adopted. In this modification, the heart-lung block was inverted with the posterior aspect facing upward, and the edge of the Petri dish was utilized as a retractor (see Fig. 3E). This maneuver enabled optimal visualization of the pulmonary venous junction, the last anatomical structure anchoring the left lung to the heart. The left pulmonary vein, bifurcating into superior and inferior branches over a short distance, was transected directly from the left atrium, preserving maximal length for cuff construction (see Fig. 3F). Complete technical details related to this step are presented in Supplementary Video 2.

### Cuff preparation

**Fig. 4:**
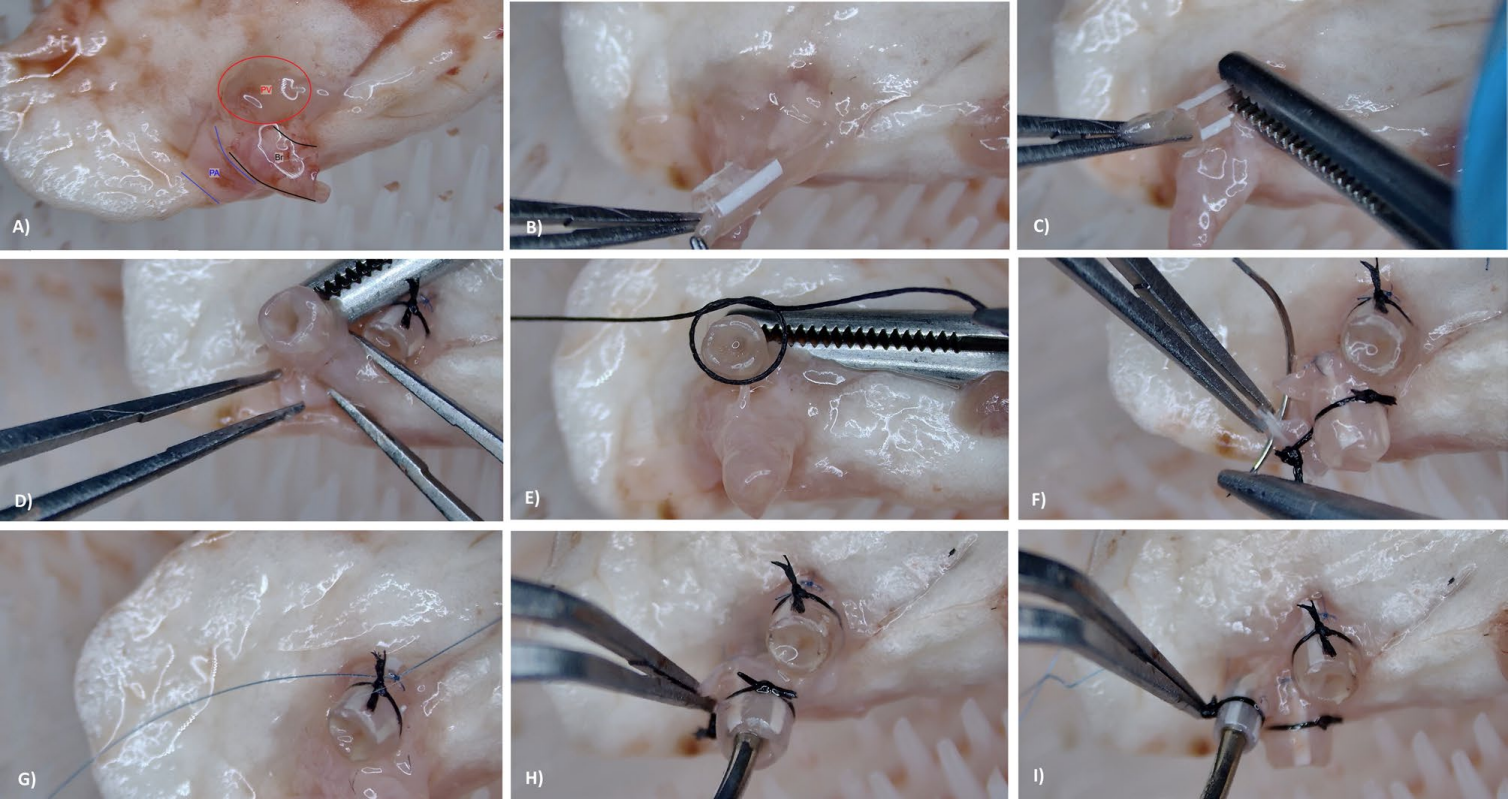
Stepwise preparation of anastomotic cuffs for donor lung implantation. (**A**) Sequential isolation of hilar structures: pulmonary vein (PV), bronchus (Br), and pulmonary artery (PA); (**B**) Insertion of each structure into its designated cuff (PV and Br with 16G; PA with 18G); (**C**) Clamping of the cuff tail with bulldog forceps to maintain orientation during preparation; (**D**) Folding the vessel or bronchial tissue back over the cuff body for circumferential coverage; (**E**) Primary fixation of the folded edge using 6 − 0 multifilament silk suture; (**F**-**G**) Additional stabilization by anchoring the distal segment of the tissue to the cuff tail using 8 − 0 monofilament Prolene^®^ suture; (**H**) Verification of bronchial lumen patency with microinstrument; (I) Verification of arterial lumen patency. Venous cuff patency was not routinely assessed due to short segmental branching. PV – Pulmonary vein; PA – Pulmonary artery; Br – Bronchus.

The explanted left lung was repositioned onto a soft-bristled brush placed within a Petri dish to facilitate atraumatic manipulation. At the hilar region, the pulmonary artery, bronchus, and pulmonary vein were gently separated from one another, and cuff preparation was sequentially performed in the following order: vein, bronchus, and artery (see Fig. 4A).

Pre-fabricated cuffs were used for all anastomoses. These measured 2.8 mm in total length (1.4 mm body and 1.4 mm tail). The arterial cuff was fashioned from an 18-gauge green intravenous catheter, while 16-gauge gray catheters were used for the bronchial and venous cuffs.

Initially, the pulmonary vein was passed through the cuff (see Fig. 4B). After securing the tail segment with a bulldog clamp (see Fig. 4C), the proximal end was folded back over the cuff body to encase it circumferentially (see Fig. 4D). The overfolded vessel wall was then fixed to the cuff using 6 − 0 multifilament silk suture (see Fig. 4E). This procedure was repeated for the bronchus and artery. Notably, for the bronchus, a small longitudinal notch was created on the cartilaginous side to facilitate eversion over the cuff.

As a novel modification introduced in this study, following primary fixation with 6 − 0 silk sutures, additional anchoring was achieved by securing the distal segment of the vessel or bronchus to the tail of the cuff using 8 − 0 monofilament (Prolene^®^) suture (see Fig. 4F and G). This step was implemented to prevent slippage of the vascular and bronchial structures from the cuff during implantation.

Finally, the lumen patency of each anastomotic cuff was assessed using an appropriately sized microinstrument (see Fig. 4H-I). Due to the short segmental branching of the pulmonary vein, lumen patency was not routinely verified for the venous cuff. A comprehensive video demonstrating the cuff preparation process is available as Supplementary Video 3.

### Recipient thoracotomy

**Fig. 5:**
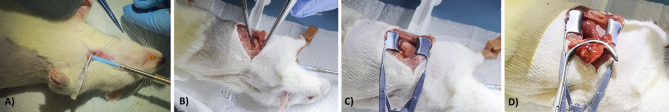
Key procedural steps of recipient thoracotomy and native lung exteriorization. (**A**) Placement of a proximal tracheostomy retraction suture (6 − 0 silk) to enable emergency airway access during lateral positioning; (**B**) Lateral decubitus positioning and performance of an anterolateral thoracotomy incision guided by the inferior scapular margin; (**C**) Insertion of a thoracic retractor for optimal exposure of the left hemithorax; (**D**) Exteriorization of the native left lung with blunt instrumentation, followed by transection of the pulmonary ligament to permit hilar dissection.

Anesthesia induction, tracheostomy-based airway access, and mechanical ventilation parameters for the recipient rat were applied identically to the donor protocol. As a procedural modification introduced in this model, to allow for emergency re-intubation in the lateral decubitus position without disturbing the surgical orientation, a 6 − 0 multifilament retraction suture was placed through the proximal end of the tracheostomy and secured externally (Fig. 5A).

A 10-mL syringe was placed beneath the right hemithorax to serve as mechanical support and maintain adequate intercostal spacing. Following identification of the inferior scapular margin, a 3-cm anterolateral thoracotomy incision was performed just below this landmark. The intercostal space was accessed via blunt and sharp dissection through the skin, subcutaneous tissue, and thoracic musculature. The ventilator was briefly paused, and the thoracic cavity was entered through the interspace with the most prominent cardiac impulse, typically the fourth intercostal space (see Fig. 5B).

Hemostasis was achieved using electrocautery, and a thoracic retractor was inserted for optimal exposure (see Fig. 5C). With the assistance of two soft-tipped instruments, the native left lung was gently exteriorized from the thoracic cavity, and the pulmonary ligament was transected (see Fig. 5D). The hilar structures were clamped, and single-lung ventilation was initiated.

To minimize the risk of right lung injury secondary to volutrauma during single-lung ventilation, a ventilator adjustment was executed: tidal volume was reduced to approximately 65% of the recipient rat’s target tidal volume. This adjustment reflects a procedural modification introduced in the present protocol.

A detailed visual demonstration of the recipient thoracotomy procedure is provided in Supplementary Video 4.

#### Implantation

**Fig. 6:**
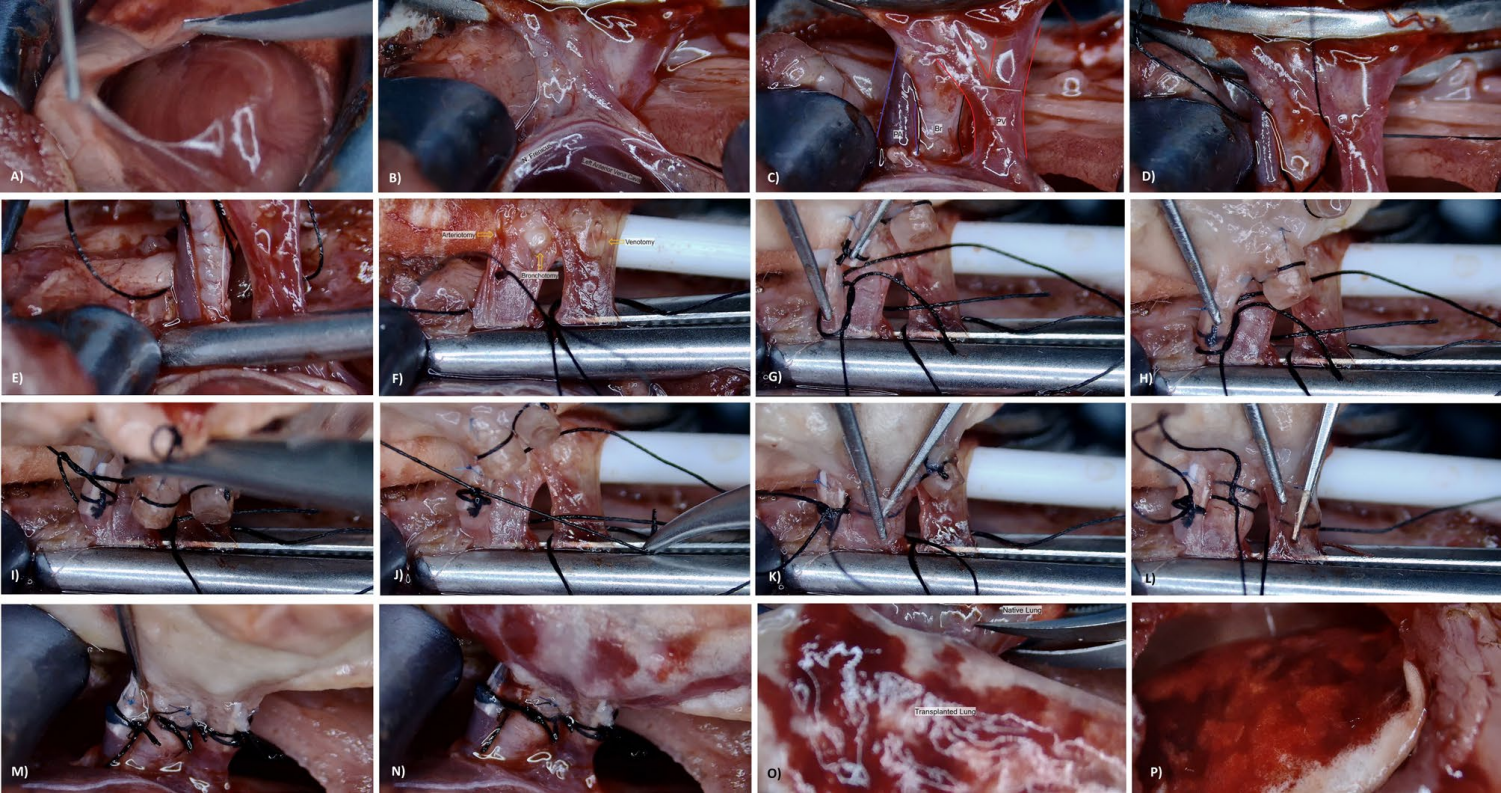
Stepwise overview of recipient implantation procedure in the rat orthotopic lung transplantation model. (**A**) Opening of the pericardial sac to expose the heart under microsurgical guidance; (**B**) Identification of the left hilar anatomy following thoracic exposure; (**C**) Hilar anatomy revealed following meticulous adjacent tissue dissection; (**D**) Pre-placement of 6 − 0 silk suspension sutures behind each hilar structure to assist cuff positioning (**E**) Application of a vascular clamp across the hilum to temporarily halt perfusion; (**F**) Recipient lumen openings created via venotomy, arteriotomy, and bronchotomy in sequence; (**G**) Identification of Prolene^®^-marked donor cuff tail as a visual guide for correct orientation during implantations; (**H**) Clamping the marked donor cuff together with the anterior arterial wall to stabilize alignment prior to final ligation; (**I**) Secure ligation of arterial cuff using the pre-positioned sutures; (**J**) Insertion of the arterial cuff and completion of the arterial anastomosis (**K**) Implantation of the bronchial cuff using the same technique; (**L**) Final implantation of the venous cuff, completing the triad of anastomoses; (**M**) Immediate transient reclamping of the pulmonary artery for 90 s following hilar clamp release; (**N**) Controlled reperfusion initiated after gradual arterial clamp release; (**O**) Excision of the native left lung to finalize graft integration; (**P**) Final appearance of a well-perfused, morphologically intact transplanted lung. PV – Pulmonary vein; PA – Pulmonary artery; Br – Bronchus.

All implantation steps were performed under 10× magnification using an operating microscope. The pericardial layer overlying the heart was incised with caution to avoid injury to the phrenic nerve (see Fig. 6A). Proceeding in a caudal-to-cranial direction, the pulmonary vein, main bronchus, and pulmonary artery were carefully dissected from adjacent structures (see Fig. 6B and C). Due to the close proximity of the artery and bronchus, an intermediate fascial plane was bluntly dissected to prevent arterial injury. Special care was also taken to preserve the integrity of the delicate venous wall by avoiding full-thickness dissection.

Once all structures were adequately exposed, 6 − 0 silk sutures, intended for fixation of the anastomotic cuffs, were pre-positioned and suspended (see Fig. 6D). The left hilar structures were then occluded using a vascular clamp aligned parallel to the vena cava (see Fig. 6E). Subsequently, venotomy, arteriotomy, and bronchotomy were performed in sequence (see Fig. 6F). Initially, the inferior and superior pulmonary veins were identified; a small notch was created using microscissors, and the venotomy was extended along the venous branches. For the arteriotomy, a transverse incision was made on the anterior surface of the pulmonary artery as close as possible to the lung parenchyma. Although the sequence of venotomy and arteriotomy was not critical, bronchotomy was deliberately performed last to prevent blood contamination of the bronchial system. Bronchotomy was carried out by making a transverse incision on the cartilaginous (anterior) surface of the main bronchus and extending it posteriorly toward the membranous wall.

During implantation, the donor lung, enclosed in moistened sterile gauze and intermittently cooled with cold preservation solution, was gently positioned atop the recipient’s native lung. Anastomoses were performed in the sequence of artery, bronchus, and vein.

The arteriotomy was opened using a clamp, and the Prolene-marked tail of the donor arterial cuff was inserted into the lumen using fine forceps (see Fig. 6G). While stabilizing the cuff inside the artery, the Prolene-marked tail was grasped together with the anterior arterial wall using a microsurgical clamp, thereby securing its position prior to ligation (see Fig. 6H). The pre-positioned 6 − 0 silk suture was tied around the artery in a manner that centered the cuff within the lumen (see Fig. 6I), completing the anastomosis (see Fig. 6J). The same sequence was followed for the bronchus and pulmonary vein (see Fig. 6K and L).

Venous anastomosis was technically the most challenging due to the short vessel length and delicate wall. A successful single-attempt anastomosis was critical to avoid laceration, particularly on the anterior wall. In such cases of minor tearing not extending to the hilar clamp, the clamp was briefly repositioned distally beneath the vena cava to enable salvage of a more intact venous segment for cuff placement.

After securing all three anastomoses, the ligation knots were trimmed, and the hilar clamp was removed to initiate reperfusion and ventilation of the graft. As a procedural refinement, immediately following removal of the hilar clamp, the pulmonary artery was transiently occluded with a blunt-tipped clamp for 60–90 s (see Fig. 6M). This maneuver enabled retrograde filling of the venous lumen by cardiac backflow, followed by gentle clamp release to ensure controlled reperfusion of the graft (see Fig. 6N). The native left lung was subsequently excised to complete graft integration (see Fig. 6O and P).

To avoid early graft injury from volutrauma, the ventilator was set to deliver approximately 85% of the target tidal volume during transition to bilateral lung ventilation. A step-by-step visual representation of the implantation technique is provided in Supplementary Video 5.

#### Thoracotomy closure, extubation, and recovery

Ventilation and perfusion of the transplanted lung were monitored intraoperatively for a brief period. Subsequently, the hilar attachments of the native left lung were transected, and the lung was carefully excised. The transplanted lung was anatomically realigned within the thoracic cavity, and thoracotomy closure was initiated. Subsequently, muscle, subcutaneous tissue, and skin layers were closed in an anatomic sequence.

**Fig. 7:**
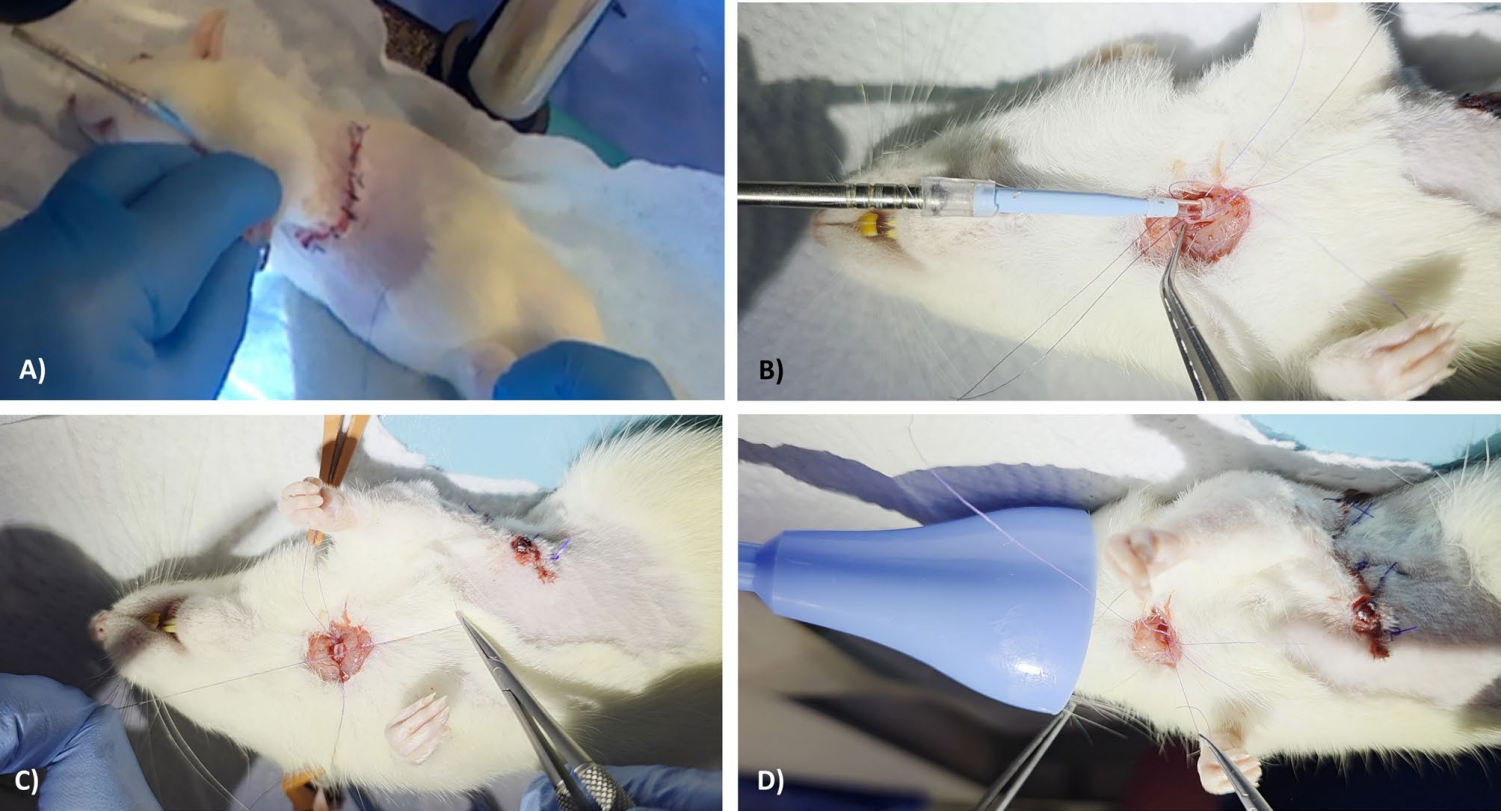
Stepwise closure of tracheostomy and postoperative respiratory support; (**A**) Supine repositioning of the recipient after anatomical thoracotomy closure; (**B**) Placement of 6 − 0 multifilament sutures through both proximal and distal tracheal margins at midline and bilateral points to prepare for tracheostomy closure; (**C**) Approximation and ligation of the anterior tracheal wall following spontaneous resumption of diaphragmatic movement; (**D**) Delivery of high-flow oxygen via a procedure-specific respiratory mask during the post-extubation phase to support recovery from anesthesia.

The recipient was then repositioned from right lateral decubitus to supine (see Fig. 7A). At this stage, the endotracheal tube was partially withdrawn from the tracheostomy to facilitate closure. The anterior wall of the trachea, previously incised during tracheostomy, was suspended using 6 − 0 Vicryl sutures placed through both proximal and distal edges at midline and bilateral lateral margins (see Fig. 7B).

Once spontaneous diaphragmatic motion resumed, extubation was performed, and the tracheostomy was secured by ligating the pre-placed sutures (see Fig. 7C). Following closure of the cervical skin incision, the recipient was repositioned in the right lateral decubitus orientation and administered high-flow oxygen (3–4 L/min) via a procedure-specific respiratory mask until full recovery from anesthesia (see Fig. 7D). All procedural steps related to thoracotomy closure and postoperative recovery are demonstrated in Supplementary Video 6.

A comprehensive summary of all procedural modifications implemented in our model and their functional advantages is detailed in Table 2 to facilitate reproducibility.

**Table 2:** Summary of technical refinements in rat orthotopic lung transplantation and their advantages.

Application / Procedural Phase	Conventional Status	New Modification	Advantages
Anostomotic cuff preparation (Before procedure)	Cuff lenght; 2 mm (body 1 mm, tail 1 mm)^6,10,13,15^	Cuff lenght; 2.8 mm (body 1.4 mm, tail 1.4 mm)	* During the cuff preparation; facilitate more secure suture placement along the cuff body * During implantation; prevents kinking of hilar structures
Anostomotic cuff preparation (Before procedure)	Cuff width; V:14G, B:14G, A:16G^13,14^	Cuff width; V:16G, B:16G, A:18G	* Sufficient lumen width for physiological flow * During implantation; reduces risk of injury to delicate vascular and bronchial structures
Application surface (Lung seperation & cuff preparation)	On a petri dish^4,7,11^	Soft-bristled brush within a Petri dish	* Protection of donor lung from compression induced injury encountered during manipulation
Pulmonary vein seperation (Lung seperation)	Anterior approach^6–8,10,11,13–15^	Dissection of the left pulmonary vein from the left atrium using a posterior approach	* Facilitating the excision of the left pulmonary vein at its longest possible length
Appropriate suture material (Cuff preparation & implantation)	6.0 / 7.0 / 8.0 monofilament suture^4,6,7,11^	6.0 multifilament suture	* Enhances mechanical strength and durability
Reinforcement of the newly structured anastomotic cuff (Cuff preparation)	Grooving the cuff with serrated instruments, Roughening the surface with sandpaper^4,13^	Securing the tissue distal to the suture to the tail of the cuff using 8.0 monofilament suture after cuff preparation	* During implantation; preventing cuff–tissue detachment caused by push–pull movements * During implantation; acts as a guide to keep the cuff within the vessel and bronchus during implantation
Utilization of procedure-specific custom-designed endotracheal tube (Donor procedure, recipient thoracotomy)	14G / 16G intravenous cannula^7,9,12^	Procedure-spesific tube is designed by combining the dilator of the central venous catheter with the infusion line	* Complete sealing of the tracheal lumen to prevent retrograde leakage of positive-pressure air * Facilitates more efficient mechanical ventilator management
Retraction suture placement (Recipient thoracotomy)	No record	Placement of a retraction suture proximal to the tracheostomy after intubation	* Facilitates intubation in the lateral decubitus position when re-intubation is required
Adjustment of the mechanical ventilator for single-lung ventilation (Recipient thoracotomy)	No Record	Initiation of ventilation with 65% of tidal volume after clamping the native left lung	* Prevents the right native lung from volutrauma-induced injury
Sequence of anastomosis (Implantation)	V-B-A / B-A-V / B-V-A formation^11,15,16^	A-B-V formation	* Adequate stabilization achieved through the initially performed arterial anastomosis * The donor lung approaching laterally into the operative field not limiting exposure for subsequent anastomoses * The most challenging venous anastomosis was facilitated by achieving sufficient stabilization of the donor lung beforehand
Implementation of controlled reperfusion (Implantation)	Release of the hilar clamp and initiation of reperfusion upon completion of implantation^7,11,14^	Temporary occlusion of the pulmonary artery for 60–90 s after releasing the hilar clamp	* Allowing adequate time for retrograde blood flow to reopen the venous lumen, thereby preventing stasis and veno-occlusive complications * Ensuring controlled reperfusion to minimize ischemia–reperfusion injury
Adjustment of the mechanical ventilator for double-lung ventilation (Implantation)	No Record	Initiation of ventilation with 85% of tidal volume after implantation	* Prevents the transplanted lung from volutrauma-induced injury
Utilization of a respiratory mask (Recovery)	No Record	Utilization of small funnels, originally intended for filling thoracic drainage bottles, as improvised respiratory masks	* Provides high-flow oxygen supplementation during the post-extubation recovery phase

### Histopathological examination

Lung tissue samples were fixed in 10% neutral-buffered formalin and embedded in paraffin to generate formalin-fixed, paraffin-embedded (FFPE) blocks. Serial sections of 4 μm thickness were obtained and subjected to hematoxylin and eosin (H&E) staining for routine morphological assessment. To evaluate collagen deposition and fibrotic remodeling, Masson’s trichrome staining was performed in accordance with the Gowari protocol. Briefly, sections were deparaffinized, rehydrated, and stained with Weigert’s iron hematoxylin to visualize nuclei, followed by aqueous ferric chloride to differentiate cytoplasmic components. Subsequent staining with phosphomolybdic-phosphotungstic acid and counterstaining with aniline blue highlighted extracellular collagen fibers. Prepared slides were then dehydrated, cleared in xylene, and mounted for light microscopic examination. Histopathological findings were semi-quantitatively evaluated based on the severity and extent of lesions, and categorized as none (absent), mild, moderate, or severe, according to the distribution and intensity of features such as alveolar congestion, peribronchial and perivascular inflammatory infiltration, and fibrosis^19^.

All histological assessments were conducted by an independent pathologist blinded to group allocation to minimize observational bias.

### Statistical analysis

Statistical analyses were conducted using SPSS software version 22.0 (SPSS Inc., Chicago, IL, USA). Descriptive statistical methods were applied to assess the normality of data distribution. Results were expressed as mean ± standard error (SE), counts, and percentages. Group comparisons of continuous variables were performed using the Mann–Whitney U test. Group comparisons of categorical variables—such as surgical success—were performed using Fisher’s exact test, given the small sample sizes and binary distribution of outcomes. Kaplan–Meier survival analysis and log-rank tests were used to evaluate early survival (Day ≥ 7) and graft survival. A p-value < 0.05 was considered statistically significant.

## Results

**Fig. 8:**
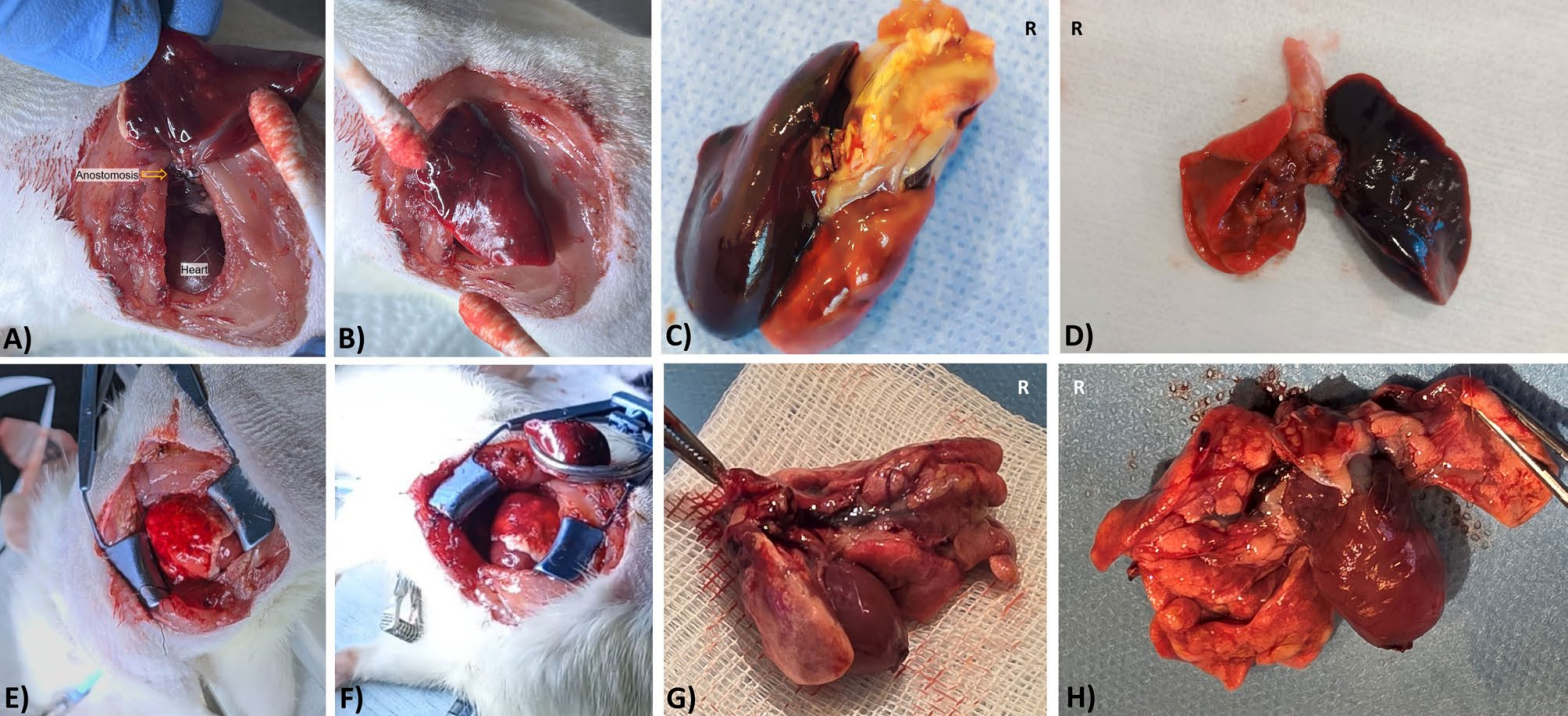
Macroscopic appearance of grafted lungs following reperfusion and sacrification in IE and RE recipients. (**A**–**B**) Intraoperative view of grafts in IE group recipients showing dark, cherry-red discoloration shortly after reperfusion; (**C**–**D**) Postmortem appearance of explanted lungs in IE recipients exhibiting hepatization features; (**E**–**F**) Well-perfused, light-red grafts in RE recipients immediately following implantation and reperfusion; (**G**) Heart–lung block at scheduled sacrification in a short-term RE survivor; (**H**) Macroscopic appearance of the heart–lung block from a six-month RE survivor showing preserved graft volume and morphology. * “R” label in the image denotes the right.

In the Initial Experience group, only 7 out of the first 13 lung transplants were completed surgically, yielding a surgical success rate of 53.8% (see Table 3). Causes of intraoperative mortality included venous laceration during anastomosis (*n* = 3), arterial rupture during bronchus-first arterial anastomosis (*n* = 1), and ventilatory failure due to endotracheal tube obstruction from secretions and/or edema (*n* = 2). In the latter cases, hemodynamic instability ensued and could not be reversed until retractor removal, repositioning to supine, and re-intubation were achieved. Among the 7 recipients in the IE group who completed the procedure and recovered from anesthesia, none survived beyond postoperative day 3. The mean survival time was 37.7 ± 9.7 h (median: 48 h; range: 6–68 h). Accordingly, early survival—defined as survival to postoperative day 7 or longer—was achieved in 0 of 7 cases (0%), indicating that short-term viability could not be sustained despite initial technical success in the Initial Experience group. In nearly all of these recipients, a striking intraoperative observation was the rapid development of a dark, cherry-red discoloration in the transplanted lung parenchyma within minutes of reperfusion (see Fig. 8A, B). During the early postoperative period, these animals exhibited hypoactivity, anorexia, a depressive posture, and progressively declining peripheral oxygen saturation.

**Fig. 9:**
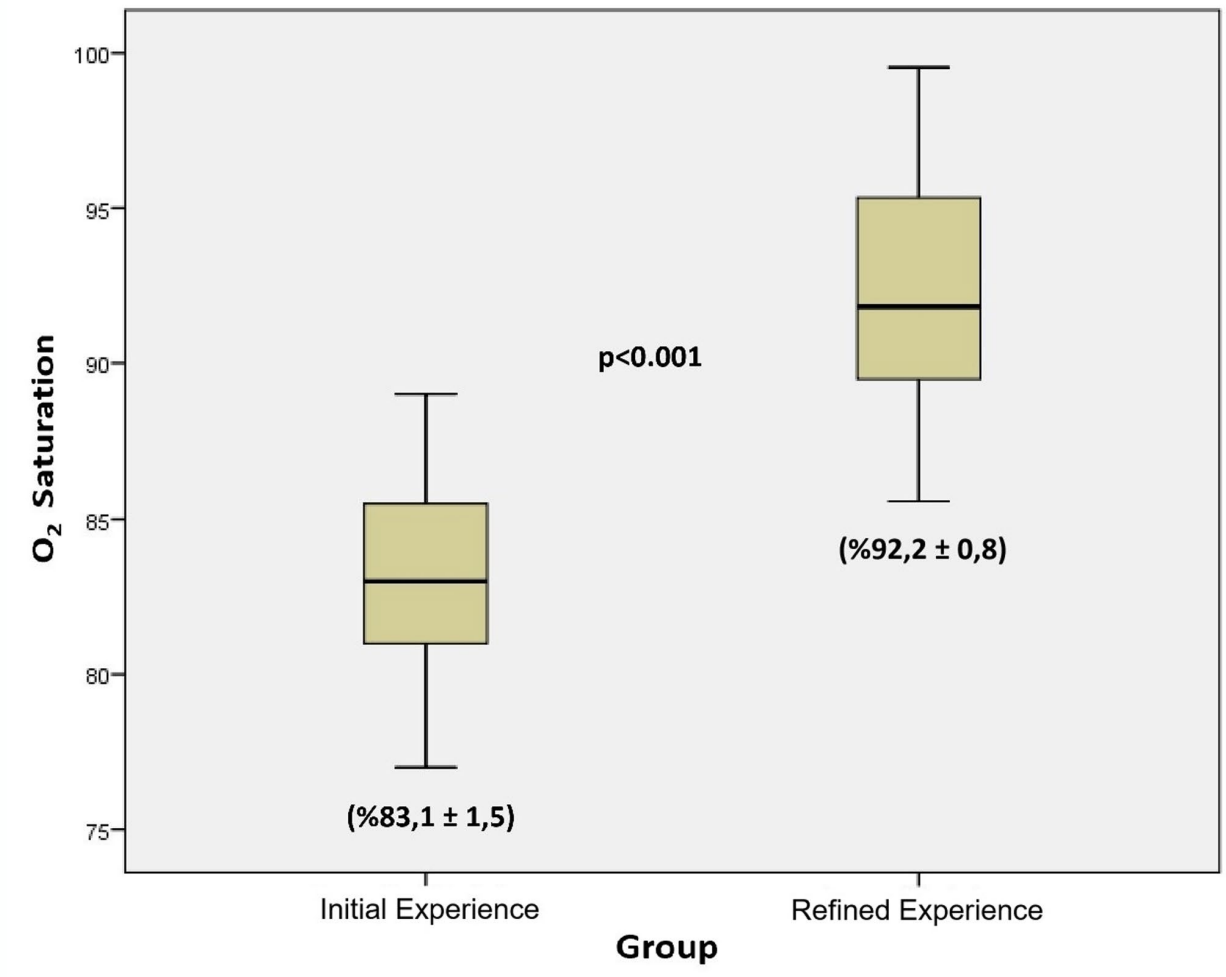
Comparison of oxygen saturation between IE and RE groups at postoperative hour 1.

In contrast, among consecutive 27 cases in the Refined Experience group—performed after sufficient procedural familiarity had been achieved and all technical modifications were fully implemented—26 procedures were successfully completed, with the exception of a single case complicated by venous laceration due to a pulmonary vein anomaly. All 26 of these recipients (100%) achieved early survival, yielding a surgical success rate of 96.3% (see Table 3). These animals exhibited light red, well-perfused, and morphologically intact grafts upon reperfusion (see Fig. 8E and F), maintained high oxygen saturation, demonstrated normal feeding and spontaneous mobility, and presented with stable clinical status. Oxygen saturation measured at postoperative hour 1 was significantly higher in the RE group (92.2 ± 0.8%) compared to the IE group (83.1 ± 1.5%, *p* < 0.001; see Fig. 9), highlighting a marked improvement in both intraoperative graft integrity and early postoperative recovery.

**Table 3:** Comparison of surgical time, surgical success, and early survival between two groups.

Variables	IE Group (*n* = 13)	RE Group (*n* = 27)	*P* value
Donor Procedure Time (min)	14.3 ± 0.6	8.4 ± 0.1	**< 0.001**
Organ Preparation Time (min)	14.4 ± 0.5	10.2 ± 0.1	**< 0.001**
Recipient Procedure Time (min)*	47.6 ± 0.9	37.1 ± 0.4	**< 0.001**
Total Procedure Time (min)*	84.8 ± 0.6	59.1 ± 0.6	**< 0.001**
Mean Survival Time (h)**	37.7 ± 9.7	Not applicable	─
Surgical success	53.8%	96.3%	**0.001**
Early survival*	0%	100%	**< 0.001**

Data are presented as mean ± SE unless otherwise stated.

* Early survival (%), recipient and total procedure time analyses were limited to cases in which implantation and thoracic closure was successfully completed (IE group: *n* = 7, RE group: *n* = 26).

** Mean Survival Time refers only to IE cases which implantation and thoracic closure was successfully completed (*n* = 7).

As expected, procedural durations were notably longer during the Initial Experience phase, when surgical familiarity was limited; however, time efficiency improved substantially with the implementation of technical refinements and increasing surgical experience (see Table 3). The most time-consuming challenges observed in the İE group included inadequate atrial margin during venous cuff preparation, difficulties in stabilizing the bronchus around the cuff, suture dislocation due to push-pull movements during implantation despite standard precautions, and disruption of one anastomosis while manipulating another in the bronchus–vein–artery or vein–bronchus–artery sequence.

Scheduled euthanasia could not be performed in the İE group, as recipients did not survive until postoperative day 7. However, seven animals that had completed the surgical procedure were examined postmortem. In nearly all of these, the transplanted lungs displayed hepatization characteristics (see Fig. 8C,D).

In the RE group, no gross pathological abnormalities were noted upon scheduled euthanasia (Fig. 8G), except for minor pleural effusion observed in 3 out of 26 cases (11.5%). In the three long-term survivors maintained for 2, 4, and 6 months respectively, body weight gain was recorded as follows: from 247 g to 363 g, 261 g to 487 g, and 250 g to 612 g, respectively. Notably, in the recipient maintained for 6 months, the native right lung had hypertrophied proportionally with somatic growth, whereas the transplanted left lung remained nearly identical in size to the original donor graft, despite appearing morphologically normal and occupying the entire left hemithorax (see Fig. 8H). Comparative hematological parameters obtained at the time of euthanasia between one-week survivors and long-term survivors are presented in Table 4.

**Table 4:** Comparison of the hemogram results of living animals across different time-based groups.

Variables	One-week Group (*n* = 23)	Long-Term Group (*n* = 3)	*P* value
WBC (thousand/µL)	7.5 ± 0.1	9.8 ± 0.6	**0.005**
Granulocyte cells (thousand/μL)	1.7 ± 0.1	1.8 ± 0.1	0.705
Lymphocyte cells (thousand/μL)	5.3 ± 0.1	7.0 ± 0.9	**0.012**
Hmg (g/dL)	14.9 ± 0.2	15.5 ± 0.3	0.275
Hct (%)	49.6 ± 0.1	47.8 ± 1.1	**0.041**
Plt (thousand/µL)	867 ± 13.8	960 ± 65	0.157

WBC: White Blood Cells, Hmg: Hemoglobin, Hct: Hematocrit, Plt: Platelets. Values are given n (%), mean ± SE.

The bold values indicate statistically significant differences between the compared groups. These values meet the predefined threshold for significance (*p* < 0.05).

**Fig. 10:**
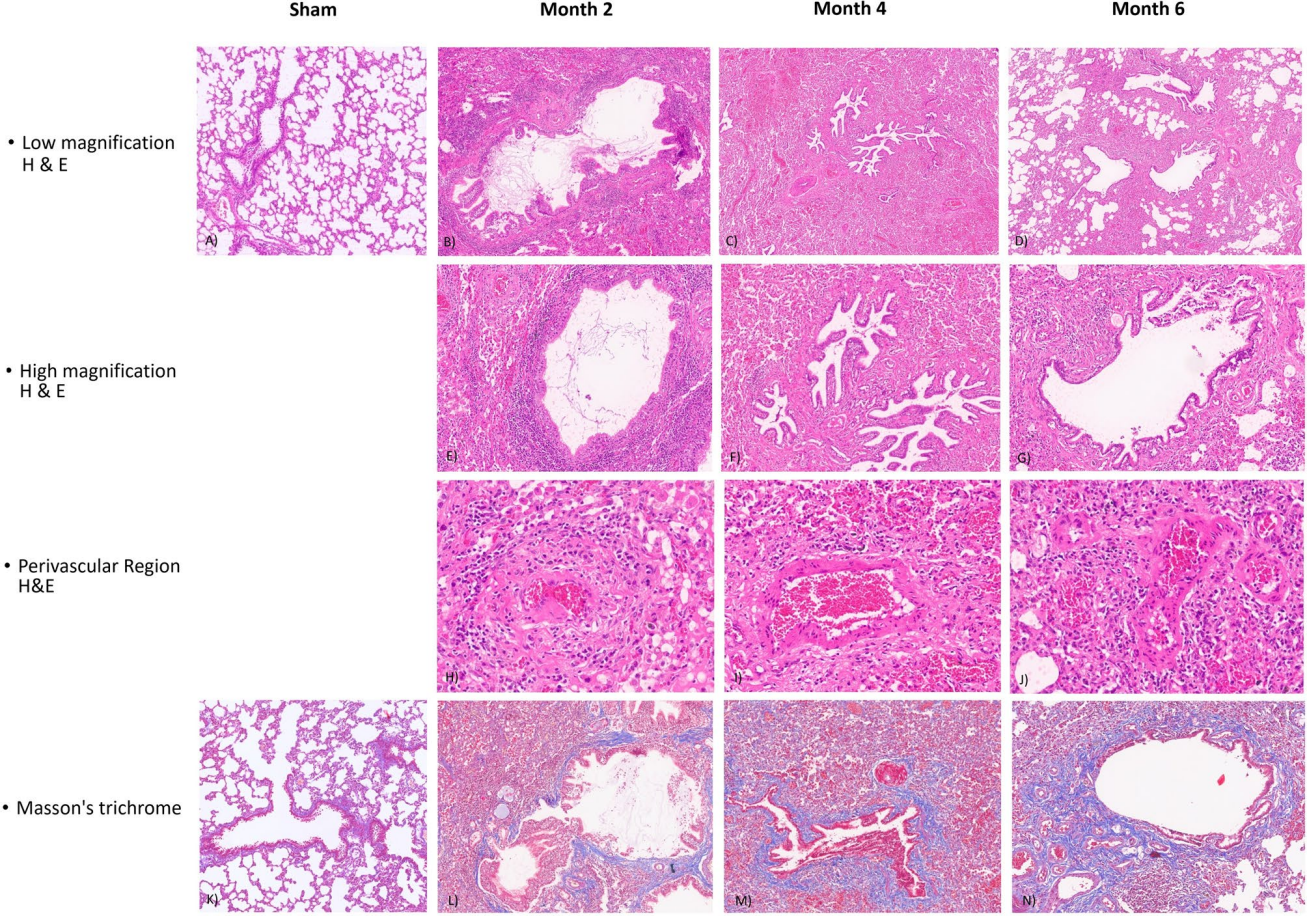
Representative histopathological features of lung grafts at different time points following transplantation and in the Sham control group. Each column corresponds to a post-transplantation time point (Sham, Month 2, Month 4, and Month 6), while each row depicts a distinct histological evaluation: low magnification H&E staining, high magnification H&E staining, perivascular region (H&E), and Masson’s trichrome staining. (**A**) Sham control: H&E-stained section showing preserved alveolar architecture with no evidence of congestion, inflammatory infiltrates, or atelectasis. (**B**–**D**) Low magnification H&E-stained sections from Month 2, 4, and 6, respectively, illustrating decreasing peribronchial inflammation and alveolar congestion over time. (**E**–**G**) High magnification H&E-stained views highlighting marked alveolar macrophage accumulation at Month 2 (**E**), minimal peribronchial infiltrates at Month 4 (**F**), and residual inflammation at Month 6 (**G**). (**H**–**J**) Perivascular H&E-stained sections demonstrating mild lymphoplasmacytic inflammation at Months 2, 4, and 6. (**K**) Sham control: Masson’s trichrome-stained section showing no collagen deposition or fibrosis. (**L**–**N**) Trichrome-stained sections from Month 2, 4, and 6, respectively, revealing gradual peribronchial and interalveolar collagen deposition consistent with evolving chronic fibrotic remodeling.

Histopathological evaluation of the Sham recipient revealed normal pulmonary architecture without any signs of pathological alterations (see Fig. 10A and K). In contrast, grafts examined at two, four, and six months post-transplantation demonstrated a time-dependent progression of chronic remodeling. At month two, hematoxylin and eosin (H&E) staining showed alveolar septal congestion and marked intra-alveolar macrophage accumulation, with peribronchial and perivascular lymphoplasmacytic infiltration, mild venulitis, and focal areas of atelectasis (see Fig. 10B and E-10H). Masson’s trichrome staining indicated early-stage fibrosis with mild peribronchial collagen deposition (see Fig. 10L). At month four, alveolar septal congestion persisted; however, inflammatory infiltration was markedly reduced (Fig. 10C and F-10I). Moderate peribronchial and mild interalveolar collagen deposition suggested progressive fibrotic change (see Fig. 10M). At month six, histological findings resembled those at month four (see Fig. 10D and G-10J), with sustained minimal inflammation and more pronounced collagen accumulation in peribronchial and interalveolar regions, indicating advancing fibrotic remodeling (see Fig. 10N).

Comparative histological analysis across time points demonstrated a progressive decline in acute rejection features—specifically alveolar congestion and perivascular/peribronchial lymphoplasmacytic infiltration—accompanied by a time-dependent increase in fibrotic alterations, characterized by collagen deposition in peribronchial and interalveolar regions, consistent with chronic rejection (see Table 5).

**Table 5:** Summary of histopathological findings at sequential time points following transplantation and in the Sham control.

Histological Variable	Sham	Month 2	Month 4	Month 6
Alveolar Septal Congestion	None	Moderate	Mild	Minimal
Alveolar Macrophage Accumulation	None	Moderate	Mild	Minimal
Peribronchial Inflammation	None	Mild to Moderate	Minimal	Minimal
Perivascular Inflammation	None	Mild	Mild	Mild
Peribronchial Fibrosis	None	Mild	Moderate	Moderate
Interalveolar Fibrosis	None	Minimal	Mild	Moderate

## Discussion

In the literature, rat lung transplantation is consistently described as an exceptionally challenging microsurgical procedure that can only be performed by highly trained surgeons operating in specialized and well-equipped laboratories^5,9,10^. Another widely noted challenge is the presence of a prolonged and steep learning curve associated with this technique^4,11^. Based on cumulative surgical experience, it has been reported that proficiency in this technique typically requires 30–50 transplantation attempts before the procedure can be completed within an optimal time frame^4,9,11^.

Our findings demonstrate that implementing specific procedural refinements, along with accumulated operator experience, substantially improved surgical success and early postoperative outcomes in this model. The Initial Experience group yielded limited procedural success and no seven-day survivors, with frequent intraoperative complications such as venous laceration or graft discoloration indicative of failed reperfusion. In contrast, the Refined Experience group—characterized by the integration of all technical modifications and enhanced procedural familiarity—achieved markedly higher surgical success (96.3%), stable recovery, improved oxygenation, and complete early survival among recipients, all within a shorter learning phase (10–15 cases). These improvements were accompanied by more reliable reperfusion, consistent graft appearance, and mitigation of previously encountered technical obstacles. Although inter-study comparisons are limited by inconsistent definitions of procedural timeframes, our time parameters fall within the expected range reported previously^4,8–12^.

Given the scarcity of long-term data in rat lung transplantation, this study contributes by characterizing the histological progression of chronic allograft remodeling in recipients surviving beyond one month. Acute rejection features—such as alveolar congestion and lymphoplasmacytic infiltration—were prominent at two months but gradually regressed, yielding to peribronchial and interalveolar collagen deposition at later time points. This transition from inflammation to fibrosis closely parallels the pathophysiology of chronic rejection in clinical lung transplantation and reinforces the biological fidelity and translational relevance of our model. The absence of such changes in sham-operated controls confirms that the observed remodeling was specific to the transplantation process. In parallel with tissue remodeling, comparative hematological assessments between one-week and long-term survivors provided additional insight. Increased granulocyte and lymphocyte counts in long-term recipients may reflect a stabilized immunological profile, whereas slightly lower hematocrit values could indicate adaptation of graft oxygenation dynamics or compensatory hematopoietic shifts. Despite the small sample size, these trends were consistent with observed clinical and histological findings, further supporting the model’s biological coherence.

From a technical standpoint, we introduced several modifications to minimize procedural complications and improve reproducibility. Departing from conventional techniques, we conducted the “lung separation” and “cuff preparation” phases on a soft-bristled brush positioned within a Petri dish. By utilizing pressure-absorbing working platforms, we protected the delicate graft from compression-related injuries.

Anastomotic cuffs are typically described as 2 mm intracatheter segments with lumen diameters of 14G, 16G, or 18G^13,14^. In contrast to most protocols, we preferred longer (2.8 mm) and narrower cuffs—18G for the artery, 16G for the vein and bronchus. We believe this design improves suture control and reduces kinking of hilar structures after implantation. The selected diameters maintain physiological flow while minimizing trauma to delicate tissues. Notably, the use of a wider cuff for the vein than the artery (16G vs. 18G) is intentional, reflecting lower venous pressures and aiming to reduce thromboembolic risk^7^.

To secure the graft within the cuffs and prevent slippage, we used 6 − 0 silk (non-absorbable, multifilament) sutures. Although finer threads like 7 − 0 or 8 − 0 are sometimes used, we found that slightly thicker multifilament sutures provided better stabilization during recipient implantation^11,14^.

A further refinement involved anchoring the tissue’s distal segment to the cuff tail with 8 − 0 polypropylene. While this added ~ 1.5 min to preparation, it offered two key advantages: (1) preventing cuff-tissue detachment during insertion, and (2) providing a visual landmark for accurate positioning. Previous strategies, such as surface roughening or grooving, were less reliable in preventing dislodgement during implantation^4^.

Tracheostomy is commonly preferred for airway access in rodent lung transplantation models due to its speed and reliability^12^. In alignment with our thoracic surgical expertise and the objective of optimizing mechanical ventilation strategies, tracheostomy was deliberately employed from the outset of our protocol. This approach was specifically intended to minimize retrograde air leakage—further supported by the use of a customized endotracheal tube designed to reduce backflow—and to facilitate a more standardized, reproducible, and clinically applicable ventilation model. These outcomes were anticipated to be more reliably achievable through tracheostomy. In contrast, orotracheal intubation is more technically demanding, time-consuming, and requires specialized equipment, although it may reduce surgical complexity^9^.

During recipient surgery—a phase requiring meticulous management—the endotracheal tube can become obstructed by secretions or pulmonary edema—complications that impair positive-pressure delivery and require urgent re-intubation. In lateral decubitus positioning, returning the animal to supine for airway access may delay intervention and precipitate hemodynamic collapse. Under these circumstances, rapid resolution is unlikely to be achieved through orotracheal intubation^8,9^. To address this critical scenario, we introduced a novel modification: a retraction suture placed through the proximal tracheostomy. This maneuver allows for immediate visualization of the trachea even in the lateral decubitus position, enabling prompt and straightforward re-intubation using a spare endotracheal tube, thereby allowing the procedure to proceed without interruption. Following this modification, no further intraoperative airway-related events were observed in our series.

Unlike most reports, we prefer an anastomotic sequence of artery–bronchus–vein^11,15–17^. For right-handed surgeons, the artery is typically the most distal and the vein the most proximal structure. Starting with the vein—being the most delicate—risks dislodgement during subsequent steps. Consequently, performing the venous anastomosis first provides insufficient stabilization of the lung, and positioning the donor lung close to the surgical field at this early stage can restrict visibility and limit instrument maneuverability. While some advocate bronchus-first anastomosis for early stabilization^11,15^, this may compromise arterial access due to close proximity of the two structures.

In our experience, initiating with the arterial anastomosis offers distinct advantages: its robust wall allows secure fixation, and lateral positioning brings the graft closer in a controlled manner without hindering subsequent steps. Completing the bronchial anastomosis next provides further stabilizes the lung, facilitating the final and most challenging step—venous anastomosis. As a complementary technical modification, following insertion of the cuffs into the recipient’s vascular and bronchial structures, we gently stabilize the Prolene-marked tail of each cuff together with the anterior wall of the structures. This ensures positional security and prevents dislodgement prior to final ligation.

Pulmonary vein anastomosis is a key determinant of success in this technically demanding procedure. Due to its immediate branching, the available length for anastomosis is shorter than that of the artery or bronchus. Similar to human anatomy, the venous wall is thinner and more delicate, making it prone to tearing even with minor technical errors. Vein rupture remains the most common cause of intraoperative mortality across studies and was likewise the most frequent complication in our series^9,18^. In cases of partial laceration of the anterior wall, we successfully employed a rescue maneuver—outlined in the surgical technique section—to complete the procedure in two early cases.

To minimize such risks, careful vein cuff preparation is essential. Given the posterior location of the left atrium and the presence of a left-sided vena cava in rats, standard anterior dissection is often limited^7–9,11,14^. Thus, we prefer a posterior dissection to maximize venous length during organ preparation phase. Therefore, we adopted a posterior dissection approach to isolate the left pulmonary vein at maximum length, enhancing cuff placement.

We consider the most critical technical refinement to be the temporary reclamping of the pulmonary artery for 60–90 s immediately after hilar clamp release. This maneuver allows controlled reperfusion and reventilation of the graft. In conventional techniques, removal of the hilar clamp permits an abrupt rush of high-pressure blood from the heart into the graft via the pulmonary artery^7,11,14^. Despite equal clamp duration, the pulmonary vein, owing to its thinner and more delicate wall, often remains collapsed, failing to establish an adequate lumen for low-pressure venous return following alveolar gas exchange. As a result, high-pressure arterial inflow encounters insufficient venous drainage, leading to blood stasis, interstitial edema, and rapid parenchymal discoloration—visibly manifesting as a cherry-red darkening of the graft within minutes. Although this may not acutely impact hemodynamics during the intraoperative period—likely due to compensatory function of the contralateral lung— it was a major contributor to poor postoperative outcomes in our initial cases. Following implementation of our modified reperfusion strategy—allowing retrograde venous filling before initiating controlled arterial inflow— all recipients achieved at least 7-day survival with markedly improved graft viability. Notably, a similar approach has been validated in a recent study demostrating long-term survival^8^.

Mechanical ventilator management proved equally critical. To our knowledge, this is the first study to describe specific ventilation strategies in this context. The custom-designed endotracheal tube used in our protocol consists of two interconnected segments. The junction between these segments acts analogously to the balloon cuff in human endotracheal tubes, achieving a complete seal of the tracheal lumen. This configuration prevents retrograde air leakage and ensures optimal efficacy of positive pressure ventilation. Given that 63% of total lung volume in rats resides in the right lung^20^, we applied targeted ventilation settings to avoid volutrauma during implantation. With the native right lung clamped, tidal volume was reduced to 65% to allow protective single-lung ventilation. Following implantation, we transitioned to double-lung ventilation and adjusted the ventilator to 85% of target volume to avoid overdistension of the newly implanted graft, which may be susceptible to edema. Unlike certain studies in the literature^8^, the use of a leak-proof entotracheal tube enabled precise pressure control during bilateral ventilation, allowing us to safely avoid high PEEP and reduce the risk of barotrauma.

Although pressure-controlled ventilation constitutes a cornerstone of perioperative management in clinical lung transplantation, its application in rodent models remains insufficiently standardized. In light of this methodological gap, we adopted a ventilation strategy guided by prevailing experimental practices and tidal volume parameters frequently cited in the literature. In the absence of formalized protocols, our approach was informed by anatomical considerations and volumetric ratios derived from published morphometric analyses, with the intent of achieving protective ventilation and mitigating graft-related injury. Notably, we referenced the lung volume distributions reported by De Backer et al.^20^ and corroborated by Lee et al.^7^, which served as the basis for our intraoperative adjustments during both single- and double-lung ventilation phases. Given the inherent fragility of rat pulmonary tissue and the paucity of precise data regarding pressure-controlled modalities in this context, we exercised caution in their implementation. Nonetheless, we recognize that future investigations may benefit from systematically evaluating these modes to enhance procedural safety, reproducibility, and translational relevance.

The main limitations of our study include the restricted number of transplants permitted due to ethical constraints, which may limit the statistical power of subgroup analyses— particularly in long-term survivors. The unavailability of inhalational anesthesia systems, which typically offer better control and faster recovery, represents another limitation; the use of general anesthesia may have contributed to intraoperative complications such as pulmonary edema and delayed awakening^21^. Suboptimal sterility and the absence of advanced postoperative care infrastructure, including intensive care units, may have further impacted early outcomes in technically successful but clinically unstable recipients. Although a single surgeon ensured procedural consistency, this introduces potential operator-related bias and limits generalizability. Furthermore, the observed short learning curve (10–15 cases) should be interpreted with caution, as it is inherently operator-dependent and may vary significantly based on individual microsurgical background and technical proficiency. Future multicenter and multi-operator evaluations are essential to confirm the technique’s generalizability and to delineate its objective learning curve. In addition, comparative studies incorporating randomized designs and literature-established techniques will be critical for a more rigorous evaluation of the method’s relative performance and reproducibility.

Despite these limitations, the high success rate and relatively short learning process—especially compared to previous models—stand out as major strengths. The incorporation of mechanical ventilation strategies, detailed accounts of perioperative challenges and their corresponding technical refinements, and long-term survival data further enhance the model’s robustness and reproducibility. Importantly, histopathological evaluation confirmed progressive chronic remodeling with declining acute rejection features, underscoring the model’s translational value and biological fidelity. Moreover, to support reproducibility and facilitate training, visual documentation and supplementary video materials were integrated into the study design, reflecting the proven educational value of such resources during the learning phase.

## Conclusion

We propose that our modified cuff technique, which encompasses perioperative processes including tailored mechanical ventilation strategies, presents itself as an optimal model for orthotopic lung transplantation in rats by addressing potential intraoperative challenges with reliable and reproducible solutions. This study also demonstrates that the model can be successfully implemented using commonly available clinical-grade materials, without reliance on specialized, high-cost infrastructure such as intensive care facilities or advanced laboratory settings. In addition to its high procedural success rate, our technique promises a short learning curve and supports long-term survival, thereby rendering it a robust platform for studies focused on chronic rejection and other long-term transplantation outcomes.

## Supplementary Information

Below is the link to the electronic supplementary material.

Supplementary Material 1

Supplementary Material 2

Supplementary Material 3

Supplementary Material 4

Supplementary Material 5

Supplementary Material 6

## Data Availability

The datasets generated during the current study are available from the corresponding author upon reasonable request.

## Declarations

### Reporting checklist

The authors have completed the ARRIVE reporting checklist.
